# Early telescopic rod osteosynthesis for Osteogenesis Imperfecta patients

**Published:** 2015

**Authors:** A Sterian, R Balanescu, A Barbilian, I Tevanov, M Carp, C Nahoi, M Barbu, A Ulici

**Affiliations:** * ”Grigore Alexandrescu” Clinical Emergency Hospital for Children, Bucharest, Romania; ** ”Carol Davila” University of Medicine and Pharmacy, Bucharest, Romania; *** ”Dr. Carol Davila” Central Military University Emergency Hospital, Bucharest, Romania

**Keywords:** telescopic rod osteosynthesis, Osteogenesis Imperfecta, Fassier Duval osteosynthesis, Lobstein disease

## Abstract

Osteogenesis imperfecta is a genetically determined pathology that implies bone variability and osteoporosis with early onset of fractures after low energy trauma. For a better understanding of the clinical problems, Sillence and Danks created a classification.

The study group consisted of 12 patients both males and females, with ages ranging from 2 years and 3 months to 12 years.

All of them came to the hospital late, after walking, after several fractures occurred and the only treatment they underwent was with prolonged cast immobilization that caused rapid bone demineralization, axial deformations of the affected bones, increased number of fractures and eventually loss of ambulation.

Following the discharged patients, we appreciated that the open bone alignment and Fassier Duval osteosynthesis were the best way to treat a patient with Lobstein disease. The results showed that by using these two techniques a lot of time is saved on a long term because all the great complications associated with older techniques are gone and a rapid ambulation is possible due to the soft tissue damage that is kept to a minimum.

## Introduction

Osteogenesis imperfecta is a genetically determined pathology that implies bone variability and osteoporosis with early onset of fractures after low energy trauma [**1**]. The two main genes involved are COL1A1 and COL1A2 and the mutations are transmitted recessively, dominant or appear spontaneously and affect the synthesis of type one collagen in a quantitative, qualitative, or mixed way [**2**]. For a better understanding of the clinical problems, Sillence and Danks created a classification that initially had 4 groups but continued to add more and more subclasses as the genetic study evolved progressing up to type XI [**3**,**4**].

**Table 1:** Sillence DO, Danks DM: The differentiation of genetically distinct varieties of osteogenesis imperfecta in the newborn period, Clin Res 26:178, 1978

Type	Inheritance	Bone fragility	Deformity of long bones	Growth retardation	Spine	Incidence
IA	Autosomal dominant	Variable, less severe than mostly	Moderate	Short stature	Scoliosis and kyphosis in 20%	1/30.000
IB	Autosomal dominant	Variable, less severe than mostly	moderate	Short stature	Scoliosis and kyphosis in 20%	1/30.000
II	Autosomal recessive	Very extreme	Crumbled bone	Unknown	-	1/62.000
III	Autosomal recessive	severe	Progressive bowing of the long bone	Severe, smallest of all patients	kyphoscoliosis	Very rare
IVA	Autosomal dominant	Moderate	Moderate	Short stature	kyphoscoliosis	unknown
IVB	Autosomal dominant	Moderate	Moderate	Short stature	kyphoscoliosis	unknown

## Materials and method

The study group consisted of 12 patients both males and females, with ages ranging from 2 years and 3 months to 12 years, with the male: female ratio of 1:2. Every patient had low trauma long bone fractures around the age of walking, the femur being the most involved. Prior to the diagnostic, the mean number of long bone fractures was 3, counting both upper and lower extremities. Every patient who was diagnosed was immediately introduced to the biophosphonates protocol. Regarding the Sillence classification, 8 patients were grouped as type III, 2 patients as type IVA and 2 patients as IB, all of whom had only an orthopaedic treatment before surgery.

All of them came to the hospital late, after walking, after several fractures occurred and the only treatment they underwent was with prolonged cast immobilization that caused rapid bone demineralization, axial deformations of the affected bones, increased number of fractures and eventually loss of ambulation. The only treatment for these cases was surgery and the method proposed was Sofield–Miller corrective osteotomies and Fassier–Duval telescopic nail osteosynthesis. Prior to the surgical intervention, patients had a bisphosphonate treatment for 7 days.

**Fig. 1:**
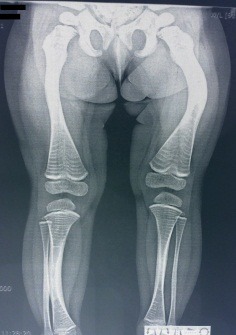
3-year-old patient with long bone bowing and multiple fractures of femur and tibia

**Fig. 2:**
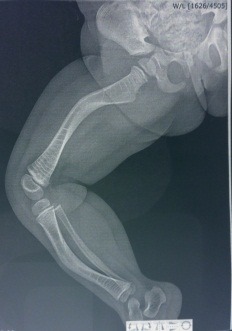
Right femur bowing after 2 fractures in the middle section and right tibia bowing after 3 fractures

**Fig. 3:**
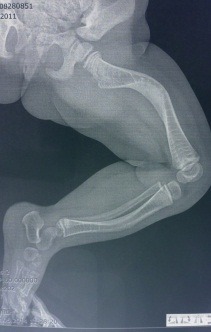
Left femur bowing after 3 fractures in the mid region and left tibia bowing after 2 fractures in the midpoint region

**Fig. 4:**
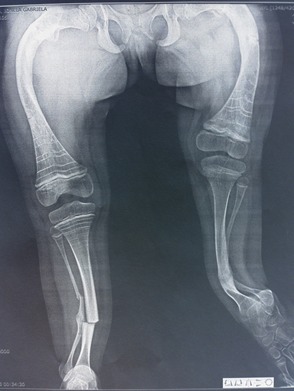
An 8-year-old patient with bilateral femur and tibia bowing treated orthopedically

**Fig. 5:**
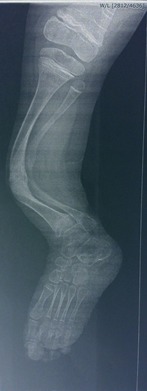
A 3-year-old patient with 4 fractures of the tibia and anterior bowing

**Fig. 6:**
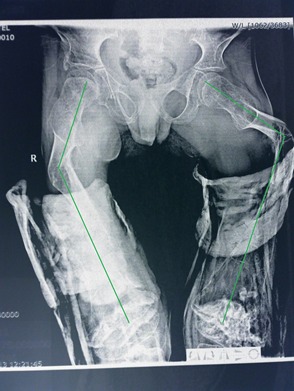
A 9-year-old patient with numerous femur fractures and almost 90° angulation

The necessity for such an aggressive surgical treatment is given by the rapidly evolving anterior bowing (sometimes associated with rotation) and the increasing incidence of the bone fracturing after cast removal. The decision of choosing Sheffield-Millard osteotomies is based on the more that 20° angulation and the narrow medullar channel that makes reaming the only solution for passing the rod. Sometimes, in very severe cases, when the distance between the epiphysis gets smaller as the bone gets more and more bowed, it is imperative to do a shorting of the segment so that the soft tissues are not put in excessive tension after alignment.

Until the development of this technique, doctors used Kirschner wire [**5**], Kuntscher nails [**6**], Rush nails [**7**], Ender nail and most recently, elastic nailing [**8**]. All the techniques had good short-term results, with good bone alignment and prevention of the refracturing of the bone. But, the biggest problem with these materials was that the patients outgrew it very fast, and problems like secondary bowing and refracturing appeared when the growth plates went too distant from the rod and the bone outgrew the nail.

Also, the low stability of the nails was a down factor, as nail loosening and slippage became a problem after the bone healed and grew in length and diameter. Regarding this fact, the first telescopic rod system was developed by Bailey and Dubow, soon after being improved to the Sheffield rod in the UK [**9**-**11**].

The basic goal of telescopic nailing was achieved with these new systems, meaning that one can obtain a long lasting osteosynthesis in a growing bone that can have good long-term results, and can decrease the risk of secondary fractures and bowing once the nail starts to tear and cannot be outgrown by the bone. The problem with these systems became clear when the children had to start moving after the surgery and the extensive soft tissue damage to the joint surface and capsule became an evident impending movement.

The main problem with these to nails is that in order to get the components in place you have to do arthorotomies and insert the nails thorough the joint cartilage [**12**-**14**]. These proved to be a problem as joint stiffness and pain prolonged the postoperative recovery period with a decrease of life quality and ambulation.

The main advantage with the Fassier Duval system is that all the maneuvers for placing the components require no arthrotomy and no joint damage. This telescopic rod achieves this by giving the operator the opportunity to insert the components in the distal epiphysis under Rx control so that the growth plate takes minimal damage and the joint surface is protected [**15**].

**Fig. 7:**
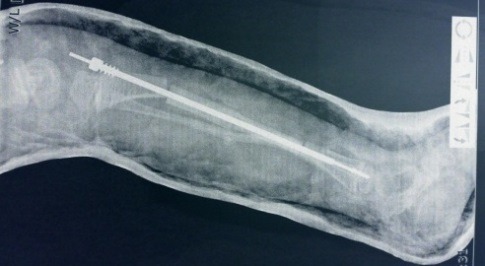
Postoperative image of a tibia – Soffield-Millar corrective osteotomies and Fasier–Duval telescopic rod osteosynthesis. Both components were introduced openly without arthrotomy of the knee or the ankle. Both components are extra articular

**Fig. 8:**
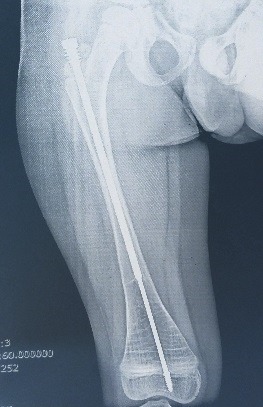
Late postoperative image of a femur that underwent alignment and osteosynthesis. The components were sliding, osteotomies were healed and no secondary bowing appeared, the system was stable and no failure, loosening or sliding of the nail happened

## Conclusion

Following the discharged patients, we appreciated that the open bone alignment and Fassier Duval osteosynthesis were the best way to treat a patient with Lobstein disease. The results showed that by using these two techniques a lot of time is saved on a long term because all the great complications associated with older techniques are gone and a rapid ambulation is possible due to the soft tissue damage that is kept to a minimum. Also, the healing of the bone and rigid system gives the child the opportunity to have a normal life without the risk of secondary fractures that could damage the nail and demand a secondary surgical intervention. The goal of the telescopic rod is to give the perfect solution for the OI patient to have a normal life without the permanent fear of having a fracture that could mean long periods of cast immobilization or surgical interventions with uncertain results.
